# 

**Published:** 1859-09

**Authors:** 


☞The subject upon which Dr. M'Quillen proposes to prepare an essay, to be read before the National Dental Society in July next at Washington City, is "The Anatomy, Physiology and Pathology of the fifth pair of nerves."

This should have filled the blank after his name, in the Minutes, on pago 120.

---

FORGETFULNESS.—Most persons are habitually forgetful on the subject of subscriptions.—After a paper or journal has been regularly sent them for one, two, three or four years, and they are finally called upon to pay, they forget that they are subscribers; they forget that they received and read the Journal; they forget that it was of any benefit, although they may have made or saved hundreds of dollars by the valuable information in its pages, while they have forgotten from whence the information came, but with self-complacent vanity imagine that all they have and all they know is the original product of their own stupendous intellects. Finally, they search the house, and discover that they have only one, two or three No's of each volume, and then declare they have received none other, FORGETTING that the Journal has been carelessly left where visitors, servants and children alike had access—some mislaid, some loaned or given away, some lost, and some destroyed. All these excuses and arguments are hatched up to escape paying the paltry sum they justly owe, and from which they have derived more benefit than could be procured by the investment of TEN TIMES the money in any other way. To the Dental periodical press are many of the ablest Dentists indebted for their success, while the Profession itself owes its ELEVATION chiefly to this INFLUENCE.

---

DR. WILLSON'S ADDRESS.—The address of Dr. E. T. Willson, of Boston, delivered before the Dental Convention at Niagara on the 5th of August, came to hand too late for the present No. of the Register. It will appear in the next.

There was a great desire expressed in the Convention that it should be put in such a form that it could be used by practitioners for distribution to their patients. In accordance with this indication, we have placed the copy in the hands of the printer, and it will be issued in a few days in convenient pamphlet form. It may be obtained at all the Dental Depots. It is a good thing, and should be in the hands of every one. T.

### CONTENTS.

Use of Collodion in exposure of Nerves.—By ABR. ROBERTSON ........ 101
Experience with Atmospheric Plates. By A. BERRY, D.D.S. ........ 104
Amalgam Work, ........ 108
Impressions, ........ 110
Dental Puffing, ........ 111
Pressure Plate. BY J. TAFT ........ 112
A Filtering Blow-Pipe ........ 114
Remarks on Porcelain Work. By M. LOOMIS, ........ 115
Fang Filling. By W. H. SHADOAN, ........ 117
Proceedings of Societies, ........ 120
Editorial, ........ 147

THE

### NEW YORK DENTAL JOURNAL,

IS ISSUED QUARTERLY.

### PRICE, $2.00 PER ANNUN, IN ADVANCE.

Each number contains sixty-four pages of matter, original and selected. A few pages of Advertisements will be received, terms as follows:

| | | | | |
|---|---|---|---|---|
| For one page, one year | | | | $25 00 |
| " | ½ | " | " | 15 00 |
| " | ¼ | " | " | 10 00 |

And for shorter periods in proportion.

All communications, subscriptions, and business notes of any kind, must be addressed to the

**"NEW YORK DENTAL JOURNAL,"**

No. 640 Broadway, New York.

